# Sociodemographic Characteristics of Pregnant Women Exposed to Domestic Violence During Pregnancy in an Iranian Setting

**DOI:** 10.5812/ircmj.11989

**Published:** 2014-04-05

**Authors:** Nayereh Azam Hajikhani Golchin, Zeinab Hamzehgardeshi, Leila Hamzehgardeshi, Mahboobeh Shirzad Ahoodashti

**Affiliations:** 1 Department of Midwifery, Islamic Azad University, Gorgan Branch, Gorgan, IR Iran; 2 Department of Midwifery and Reproductive Health, Shahid Beheshti University of Medical Sciences, Tehran, IR Iran; 3 Department of Midwifery, Mazandaran University of Medical Sciences, Sari, IR Iran; 4 Department of Family Health, Public Health Center, Mazandaran University of Medical Sciences, Sari, IR Iran; 5 Department of Internal Medicine, Mazandaran University of Medical Sciences, Sari, IR Iran

**Keywords:** Pregnant Women, Domestic Violence, Pregnancy, Iran

## Abstract

**Background:**

Domestic violence refers to any type of physical, sexual, and psychological abuse enforced in the setting of familial relationships. Domestic violence has a significant relationship with poor outcome among pregnant women. Success in resolving this social phenomenon rests on accurate assessment of the society and the factors associated with violence in that specific community.

**Objectives:**

The present study was conducted to assess the demographic characteristics of pregnant women exposed to different types of domestic violence during pregnancy in Iranian setting.

**Patients and Methods:**

This is a descriptive-analytic, cross-sectional study. Sampling was done with convenience sampling method. in the current study, 301 pregnant women aged 15-45 years of Iranian nationality who were referred to the hospital for delivery or abortion, regardless of the gestational age, were selected as the subjects. Data collection tools consisted of a sociodemographic questionnaire and a violence checklist. Violence was assessed using Revised Conflict Tactics Scale (CTS2). Data were analyzed using descriptive and analytic statistics on SPSS version 16 (SPSS, Chicago, IL, USA) and STATA version 10. The characteristics of the participants were presented as mean ± SD or number and percentage. Differences between variables were determined by the χ2 test, and multivariate logistic regression. P < 0.05 was considered significant.

**Results:**

According to the findings, 34.56% of participants had experienced psychological violence, 28.24% physical violence, and 3.65% sexual violence. Multivariate logistic regression revealed a statistically significant relationship only in the case of physical violence and history of penal conviction for partner (Adjusted Odds Ratio (AOR) = 12.60) and a patriarchal household (AOR = 16.75).

**Conclusions:**

As domestic violence is greatly influenced by the customs and cultures of each community, no single strategy can be adopted to resolve it universally. Simultaneously, it is necessary to adopt comprehensive measures to control factors associated with domestic violence in the healthcare, judiciary, and the educational systems in order to prevent and curb this social challenge.

## 1. Background

Domestic violence refers to any type of physical, sexual, emotional or verbal abuse enforced in the setting of familial relationships (1). Women are susceptible to violence throughout their lifespan, but this is most common in their fertile years (2). According to the reports of the World Health Organization (WHO), 16%-52% of women experience violence at the hand of their sexual partner. Around 28% of women in developed countries and 18%-67% of those in developing countries report at least one event of physical abuse (3). Domestic violence may occur, intensify, or alleviate during pregnancy due to a number of reasons including incorrect opinions of pregnancy, abnormal emotions of the partner regarding pregnancy, and the reduction in sexual contact (4). Recent studies indicate that in general, 4%-37% of women experience violence during pregnancy while a considerable fraction of them did not experience it before they became pregnant (5).

A cross sectional study on 1379 pregnant Brazilian women reported 19.1% verbal abuse and 6.5% physical and sexual abuse (6). In Tehran, the prevalence of domestic violence against pregnant women is reportedly 60.6%, consisting of 60% emotional violence, 14.6% physical violence, and 23.5% sexual violence (7). According to a study in Durban, South Africa, the risk of mental disorders in pregnant women rises by 1.5 fold with each occurrence of emotional abuse, and by 2-fold with each event of sexual abuse (8). The complications of violence in pregnancy include abortion, preterm labor, low birth weight, chorioamnionitis, low Apgar score on birth, and C-section (9). A study in Brazil demonstrated a significant relationship between emotional violence in pregnancy and premature rupture of membranes, urinary infections, headache, and high risk sexual behaviors. Moreover, a significant relationship was found between physical and sexual violence in pregnancy and loss of libido, premature rupture of membranes, urinary infections, and vaginal bleeding (10). Studies in different countries indicate that the demographic factors associated with violence vary from one society to another. While some factors may promote violence in a community, they may protect against violence in another (11). Success in resolving this social phenomenon rests on accurate assessment of the society and the factors associated with violence in that specific community.

## 2. Objectives

The present study was conducted to assess the demographic characteristics of pregnant women exposed to different types of domestic violence during pregnancy in Deziani Labor Clinic in Gorgan, Iran, so that the results may help us identify the current situation and contribute to the appropriate planning for dealing with this social challenge.

## 3. Patients and Methods

 It was a descriptive-analytic, cross-sectional study conducted from March.1.2011 to September.31.2012, in which participated 301 Iranian pregnant women aged 15-45 years who were referred to Deziani Labor Clinic in Gorgan, Iran, for delivery or abortion, regardless of the gestational age. The Participants of the study were selected through convenience sampling. The other inclusion criteria were at least three years of married life, and lack of known physical or mental disorders. Exclusion criteria were individuals with poor obstetric history in previous pregnancy and individuals with known chronic diseases. With regard to the type 1 error (alpha) of 5%, and accuracy of 0.5, and the most common domestic violence among pregnant women (psychological violence) of previous researches that was 25.6% (12), the population was estimated as 300 subjects .

Observing the ethical issues, the trained interviewers questioned the participants in a confidential setting. The questionnaires addressing abuse and sociodemographic characteristics were completed by the interviewers in a strictly private environment. Data collection tools consisted of a sociodemographic questionnaire and a violence checklist. The former dealt with the demographic characteristics of the women and her partner. Violence was assessed using Revised Conflict Tactics Scale (CTS2). Affirmative answer to each question denoted violence. This instrument has been applied in many countries with a variety of cultures (9). It has been evaluated for reliability and validity in Iranian culture, yielding a repeatability correlation coefficient of 0.9 (13).

Data from 301 questionnaires were analyzed using descriptive and analytic statistics on SPSS version 16 and STATA version 10. Overall, there were 10 questionnaires (3%) that had more than 20% unanswered questions which were excluded from the study and replaced by new ones. Response rate was above 80%. In order to study the association between domestic violence (physical, psychological and sexual) and the couples’ characteristics, the individuals were classified as either abused (those with at least one affirmative answer to the physical/sexual/psychological screening questions) or non-abused (those who gave no affirmative response to the screening questions). The couples’ characteristics were compared between these two groups. The normal assumption of data was checked. Chi-square test and crude odds ratio was used for univariate analysis. Also, multivariate logistic regression was used to adjust the analysis. Hence, physical, psychological, and sexual violence were considered as the dependent variable and marriage duration, ethnicity, unwanted pregnancy, smoking partner, drug abuse and history of penal conviction for partner, interferences of the partner’s family, a patriarchal household, husband’s ill temper, history of violence in childhood, and parity were considered independent variables. In addition, student t-test was used to compare continuous variables in the two groups. P values < 0.05 were considered significant. In addition, variables which yielded P values < 0.2 on univariate analysis entered the multivariate logistic regression model. All participants were informed about the goals and procedure of the study. Furthermore, they were assured that they could voluntarily participate in the study, and that they could refuse to continue their participation any time they wished. Besides, they were sure that the data would be confidential and their identity would not be revealed in any steps of the study. Finally, all participants signed written consent to participate in the study. The Ethical Protocol was approved by the Research Deputy of Islamic Azad University Gorgan branch, Gorgan, IR Iran (code number: 1556-89/11/24).

## 4. Results

### 4.1. Characteristics of the Abused and Nonabused Pregnant Women

The abused women had a mean age of 26.34 ± 4.62 years, and the non-abused had a mean age of 26.21 ± 5.73 years (Table 1). According to the findings, 34.56% of participants had experienced psychological violence, 28.24% physical violence, and 3.65% sexual violence. The mean ages of the abused and nonabused women were not significantly different for different types of abuse. Moreover, the mean ages of partners in the two groups were not significantly different for different types of abuse (Table 2). The results of bivariate analysis revealed statistically significant relationships between physical violence and marriage duration of 6-10 years compared to 11 years or more (crude odds ratio (COR) = 2.40), unwanted pregnancy (COR = 2.58), smoking partner (COR = 2.33), drug abuse by partner (COR = 5.30), history of partners' penal conviction (COR = 14.53), interferences of partner’s family (COR = 2.73), a patriarchal household (COR = 3.54), history of violence in childhood (COR = 2.73), husband’s ill temper (COR = 2.60), and multiparous wife (COR = 2.12) (Table 3).

**Table 1.** Characteristics of the Abused and Nonabused Pregnant Women ^a^

Characteristics	Abused Women (n = 200)	Non Abused Women (n = 101)	P Value
**Continues variables**			
**Age, y**	26.34 ± 4.62	26.21 ± 5.73	> 0.05
**Husband’s age, y**	30.37 ± 6.17	29.82 ± 6.47	> 0.05
**Duration of marriage**	6.44 ± 3.83	6.27 ± 4.90	> 0.05
**Discrete variables**			
**Marriage duration, y**			< 0.05
≥ 11	23 (11.5)	23 (22.8)	
6-10	52 (26)	27 (26.7)	
1-5	125 (62.5)	51 (50.5)	
**Ethnicity**			> 0.05
Fars	106 (53)	42 (41.6)	
Zaboli	68 (34)	36 (35.6)	
Turkmen	19 (9.5)	16 (15.8)	
Cossack	7 (3.5)	7 (7)	
**Unwanted pregnancy**			
No	176 (88)	99 (98)	
Yes	24 (12)	2 (2)	< 0.05
**Smoking partner**			< 0.05
No	166 (83)	96 (95)	
Yes	34 (17)	5 (5)	
**Drug abuse by partner**			< 0.01
No	184 (92)	98 (97)	
Yes	16 (8)	3 (3)	
**History of Partners' penal conviction**			< 0.001
No	184 (92)	101 (100)	
Yes	16 (8)	0 (0)	
**Interferences of partner’s family**			< 0.05
No	166 (83)	99 (98)	
Yes	34 (17)	2 (2)	
**A patriarchal household**			< 0.001
No	152 (76)	98 (97)	
Yes	48 (24)	3 (3)	
**Husband’s ill temper**			< 0.01
No	158 (79)	98 (97)	
Yes	42 (21)	3 (3)	
**History of violence in childhood**			< 0.01
No	153 (76.5)	99 (98)	
Yes	46 (23.5)	2 (2)	
**Parity**			< 0.01
Nulliparous	118 (59)	53 (53.5)	
Multiparous	82 (41)	48 (47.5)	

^a^ Data are presented in Mean ± SD or No. (%).

**Table 2.** Prevalence of the Physical/Psychological/Sexual Domestic Violence Among Pregnant Women (n = 301) ^a^

Type of Violence	Prevalence
**Physical Violence**	85 (28.24)
**Psychological Violence**	104 (34.56)
**Sexual Violence**	11 (3.65)
**Abused women**	200 (66.45)
**Non**a**bused women**	101 (33.55)
**Total**	301 (100)

^a^ Data are presented in No. (%).

**Table 3.** Related Factors of the Physical Domestic Violence Among Pregnant Women ^a^

Variables	(COR, 95% CI)	(AOR, 95% CI)
**Marriage duration, y**	-	-
≥ 11	1 (ref)	-
6-10	2.4 (1.07-5.4) ^b^	-
1-5	0.99 (0.46-2.11)	-
**Ethnicity**	-	-
Fars	1 (ref.)	-
Zaboli	0.65 (0.26-1.6)	-
Turkmen	0.87 (0.09-8.55)	-
Cossack	1.15 (0.67-2)	-
**Unwanted pregnancy**	-	-
No	1 (ref.)	-
Yes	2.58 (1.13-5.91) ^b^	-
**Smoking partner **	-	-
No	1 (ref.)	-
Yes	2.33 (1.16-4.68) ^b^	-
**Drug abuse by partner**	-	-
No	1 (ref.)	-
Yes	5.30 (1.89-14.84) ^c^	-
**History of partner's penal conviction**	-	-
No	1 (ref.)	1 (ref.)
Yes	14.53 (3.11-67.83) ^d^	12.60 (2.52-63.04) ^c^
**Interferences of partner’s family**	-	-
No	1 (ref.)	-
Yes	2.73 (1.31-5.69) ^b^	-
**A patriarchal household**	-	-
No	1 (ref.)	1 (ref.)
Yes	3.54 (1.89-6.63) ^d^	16.75 (1.81-155) ^b^
**Husband’s ill temper**	-	-
No	1 (ref.)	-
Yes	2.60 (1.34-5.03) ^c^	-
**History of violence in childhood**	-	-
No	1 (ref.)	-
Yes	2.73 (1.44-5.17) ^c^	
**Parity**	-	
Nulliparous	1 (ref.)	-
Multiparous	2.12 (1.27-3.53) ^c^	

^a^ Abbreviations: AOR, adjusted odds ratio; CI, confidence intervals; COR, crude odds ratio.

^b^ P value < 0.05.

^c^ P value < 0.01.

^d^ P value < 0.001.

It is noteworthy that multivariate logistic regression revealed a statistically significant relationship only in the case of physical violence and history of penal conviction for partner (Adjusted Odds Ratio (AOR) = 12.60), and a patriarchal household (AOR = 16.75) (Table 3). Bivariate analysis indicated statistically significant relationships between psychological violence and marriage duration of 1-5 years compared to 11 years or more (COR = 2.36), unwanted pregnancy (COR = 6.67), smoking partner (COR = 3.27), history of penal conviction for partner (COR = 1.59), interferences of partner’s family (COR = 10.59), a patriarchal household (COR = 10.60), history of violence in childhood (15.71), and wife’s ill temper (COR = 2.45) (Table 4). The results of bivariate analysis demonstrated statistically significant relationships between sexual violence and partner’s alcoholism (COR = 9.04), history of partner's penal conviction (COR = 10.54), and unemployed partner (COR = 4.91) (Table 5).

**Table 4.** Related Factors of the Psychological Violence Among Pregnant Women

Variables	(COR, 95% CI)
**Marriage duration, y**	
≥ 11	1 (ref.)
6-10	1.57 (0.75-3.28)
1-5	2.36 (1.22-4.57) ^a^
**Ethnicity**	
Fars	1 (ref.)
Zaboli	1.67 (0.15-18,87)
Turkmen	3.89 (0.98-16.97)
Cossack	1.07 (0.14-9.39)
**Unwanted pregnancy**	
No	1 (ref.)
Yes	6.67 (8.53-28.70) ^b^
**Smoking partner**	
No	1 (ref.)
Yes	3.27 (1.32-8.10) ^a^
**History of penal conviction for partner**	
No	1(ref.)
Yes	1.59 (1.45-1.73) ^a^
**Interferences from partner’s family**	
No	1 (ref.)
Yes	10.59 (2.49-45.08) ^c^
**A patriarchal household**	
No	1 (ref.)
Yes	10.60 (3.21-35.01) ^c^
**Wife’s ill temper**	
No	1 (ref.)
Yes	2.45 (1.44-4.15) ^b^
**History of violence in childhood**	
No	1 (ref.)
Yes	15.71 (3.73-66.18) ^c^

^a^ P value < 0.05.

^b^ P value < 0.01.

^c^ P value < 0.001.

**Table 5.** Related Factors of the Sexual Violence Among Pregnant Women

Variables	(COR, 95% CI) ^a^
**Ethnicity**	
Fars	1 (ref.)
Zaboli	8.32 (0.88-83.02)
Turkmen	2.33 (0.11-3.23)
Cossack	3.56 (0.42-30.06)
**Unwanted pregnancy**	
No	1 (ref.)
Yes	0.9 (0.11-7.29)
**Smoking partner**	
No	1 (ref.)
Yes	1.47 (0.31-7.14)
**Drug abuse by partner**	
No	1 (ref.)
Yes	3.82 (0.76-19.16)
**Partner’s alcoholism**	
No	1 (ref.)
Yes	9.04 (2.64-32.75) ^b^
**History of penal conviction for partner**	
No	1(ref.)
Yes	10.54 (2.43-45.79) ^b^
**Interferences from partner’s family**	
No	1 (ref.)
Yes	1.74 (0.36-8.42)
**A patriarchal household**	
No	1 (ref.)
Yes	3.06 (0.86-10.86)
**Wife’s ill temper**	
No	1 (ref.)
Yes	0.34 (0.1-1.21)
**Husband’s ill temper**	
No	1 (ref.)
Yes	2.36 (0.6-9.28)
**History of violence in childhood**	
No	1 (ref.)
Yes	2.17 (0.51-12.1)
**Husband's job**	
Employed	1 (ref.)
Unemployed	4.91 (1.95-25.31) ^c^

^a^ Crude Odds Ratio, 95% confidence intervals

^b^ P value < 0.001.

^c^ P value < 0.05.

## 5. Discussion

In the current study, 34.56% of participants had experienced psychological violence, 28.24% physical violence and 3.65% sexual violence. This was almost consistent with findings reported by Jahanfar in Tehran (7). The prevalence of violence varies in different societies, ranging from 4% in urban Japan to 71% in Ethiopia (14). The discrepancy seems to reflect the different degrees of inclination among women to reveal their violent experiences, as well as differences in culture and interviewers’ skills (15). Psychological violence has been the most prevalent type in most studies. It appears that cultural changes tend to reduce physical violence and increase psychological violence. Previous studies indicated that psychological violence imposes greater pan than physical or sexual violence (7, 16, 17).

Sexual violence was the least frequent type reported. Sexual violence reflects very intense violence (18). Previous studies suggest that the most intense psychological violence leads to sexual abuse (19). The low rate of sexual violence in the current and other similar studies may be due to the fact that timidity renders women unable to recount their experience of sexual violence, as well as the unquantifiable nature of this type of violence (20). In addition, the fact that sexual violence leaves no external clue makes it more hidden (7). Finally, some cultures may not consider forced sexual contact between married people as rape, while psychologists refer to it as intermarriage rape or domestic rape (21).

According to the findings of the current study, no significant relationship was found between women’s age and their experience of psychological, physical and sexual violence, which is in line with findings of Rador (22). This indicates that women of any age are at risk of violence. Nevertheless, many studies reported younger women (11) or older women (23) to be more susceptible to violence. According to the other studies, although the occurrence of violence increases with advancing age of women, the rate of reporting these events tends to fall, as older women are often not only wives, but also mothers, and are thus less likely to report violence (24). The findings of the present study indicated no significant relationship between man and woman’s education and occurrence of violence. In a study by the World Health Organization, inequality of education was found to be a major cause of violence (11). It must be noted that the traditional patriarchal culture obligates even educated women to accept men’s authority in order to avoid divorce and its impact on children. This study did not find a significant relationship between different types of violence and men’s polygamy or women’s previous marriage, which is inconsistent with many (11, 15). This may be due to the small number of polygamous men among the current study subjects.

The current study found a significant relationship between patriarchy and physical and psychological violence, which is in line with previous studies. A Nigerian study reported that women whose behavior is controlled by their husband are four times more likely to experience violence, whereas women with independent decision making power, and those with a deciding power equal to their husbands reported fewer cases of violence (25). In traditional patriarchal communities, women’s justification of their partners’ violence is strongly correlated with the occurrence of violence (26). A study on 600 Chinese women reported the lifelong prevalence of physical and sexual violence to be 43% (27). Violence occurred more frequently in women who justified their husband’s use of force. Examples of statements used by these women include: “It is important that a man should make his authority clear to his wife”, or “A good wife is supposed to obey her husband at all times”. The findings indicate that history of violence in childhood is significantly related to physical and verbal abuse. Girls who observe their mother abused by their father are more likely to assume that violence is natural in their own married life (14, 18). Similarly, men who observed their parents’ violence are more likely to neglect their wives’ rights (11, 28). The findings of the current study indicated a significant relationship between unwanted pregnancy and verbal or physical abuse, which was consistent with previous studies (11, 14). Violence affects the mind and body, weakening sexual autonomy and compromising the power to make decisions about pregnancy (29). A Tanzanian study reported that violence renders women unable to discuss the choice of contraceptive drugs with their husbands (30). As Iranian families tend to have fewer children, unwanted pregnancies raise tensions in household and bring about the settings for violence. The current study found the use of cigarettes and narcotics by partners to be significantly associated with verbal and physical violence, consistent with findings of previous studies (11, 15). It must be noted that the personal characteristics of addicts lead to behaviors which make relationship with their partners difficult.

According to the findings of the current study, there is a significant correlation between interferences by the partner’s family and frequency of physical and verbal violence. It appears that interventions of the others, especially if they live together, may cause household problems and violence (1). The current study indicates a positive relationship between marriage duration and physical and verbal violence, which is in line with the previous reports (16). A longer marriage is associated with improved understanding and knowledge of the partner’s personality, which enhances the couple’s relationship. The current study findings suggest a significant relationship between partner’s penal conviction and all three types of violence, consistent with the findings of many other studies (11, 15, 16). According to the current study findings, woman’s ill temper is significantly associated with verbal violence, while man’s ill temper is significantly associated with physical violence. The ill-tempered people tend to keep finding faults with the other person, facilitating aggressive behaviors.

In addition, a significant relationship was found between parity and physical violence. This may be caused by the financial burden imposed by the increasing number of children, leading to tension in the household. Moreover, the partner’s unemployment was found significantly associated with sexual violence. As unemployed men tend to spend more time at home, the frequency of sexual violence is higher among them. This study found that alcoholism is significantly associated with sexual violence only. As aggression is an immediate effect of alcohol use, it puts the reasoning process out of balance and prepares the person for violent behavior.

The present study had certain limitations. Firstly, since it was a cross-sectional study, there were limitations in identifying causal relationships. Secondly, collection of data related to domestic violence entails several methodological challenges, including recall bias, and sensitivity of questions. In addition, the differences in cultural and social norms regarding the social undesirability of domestic violence increase the risk of underreporting. As domestic violence is greatly influenced by the customs and cultures of each community, no single strategy can be adopted to resolve it universally. What is certain, as outlined in the declaration of human rights (1997, Vienna), “Men need to change their attitude towards women, and women need to help men revise their opinion of women”. Simultaneously, it is necessary to adopt comprehensive measures to control factors associated with domestic violence in the healthcare, judiciary, and the educational systems in order to prevent and curb this social challenge.
